# Global, Regional, and National Burden of Tracheal, Bronchus, and Lung Cancer in 2022: Evidence from the GLOBOCAN Study

**DOI:** 10.3390/epidemiologia5040053

**Published:** 2024-12-17

**Authors:** Rajesh Sharma, Jagdish Khubchandani

**Affiliations:** 1 Department of Humanities and Social Sciences, National Institute of Technology Kurukshetra, Kurukshetra 136119, India; rajesh.sharma@nitkkr.ac.in; 2 Department of Public Health Sciences, New Mexico State University, Las Cruces, NM 88003, USA

**Keywords:** tracheal, bronchus, lung cancer, incidence, mortality, smoking, air pollution

## Abstract

Background: Tracheal Bronchus and Lung cancers (TBL) represent one of the leading causes of cancer deaths worldwide. This study aimed to examine the disease and economic burden of TBL cancers in 185 countries worldwide in 2022. Methods: The estimates of TBL cancer incidence and mortality (counts and age-standardized rates) were obtained from the GLOBOCAN 2022 data produced by the International Agency for Research on Cancer. Mortality-to-incidence ratios (MIR) were utilized as a proxy of 5-year survival rates. Multivariate regression was utilized to examine the association between TBL cancer burden and tobacco use prevalence. Results: Globally, there were 2.48 million incident cases and 1.82 million deaths due to TBL cancers in 2022. Males accounted for 63.4% of incident cases (1.57 million) and 67.85% of TBL deaths (1.23 million) in 2022. For both sexes combined, the age-standardized rate was 23.1 per 100,000, and the age-standardized mortality rate was 16.8/100,000. The Mortality-to-incidence ratio (MIR) at the global level stood at 0.71. Eastern Asia had the largest burden of TBL cancers among the 21 UN-defined regions, with around 51% of incident cases (1.24 million) and 46.9% of global deaths (851,876), followed by Northern America (incidence: 257,284; deaths: 150,675) and Eastern Europe (incidence: 158,141; deaths: 126,840). At the country level, human development index (HDI) and adult tobacco use prevalence could explain 67% and 64% variation in ASIR and ASMR, respectively. HDI was statistically significantly related to MIR, explaining a 48% variation in MIR. Conclusions: With 1.9 million deaths in 2022, TBL cancer is a significant global cause of mortality. Despite the knowledge and awareness of smoking and lung cancer, adult smoking rates remain high in many countries, including the United States and China. Renewed and sustained global efforts are needed to reduce smoking prevalence and PM2.5 levels, particularly in China and low- and middle-income countries.

## 1. Introduction

Tracheal, Bronchus, and Lung (TBL) cancers are among the leading causes of cancer-related morbidity and mortality worldwide. In 2019, TBL cancer accounted for 2 million deaths and 2.3 million new cases globally [1]. According to GLOBOCAN 2020, East Asia was the most affected region, responsible for nearly 46% of the global lung cancer incidence in 2020 [2]. Tobacco smoking, air pollution, and environmental factors such as arsenic, nickel, radon, X-rays, gamma radiation, and asbestos are recognized lung cancer risk factors [3]. Lung cancer has low survival rates, particularly non-small cell lung carcinomas (NSCLC), which constitute 85–90% of lung cancer cases and are often diagnosed at advanced stages, resulting in poor prognosis. Recent SEER statistics indicate that the 5-year survival rate for lung cancer in the United States is approximately 25% [4], which remains even lower in developing countries.

As a major public health challenge, the epidemiological profile of TBL cancer must be examined from time to time. The IARC has previously released cancer estimates for 2002, 2008, and 2018 [5,6,7]. However, there are no published studies on the global estimates for TBL cancers after the acute phase of the COVID-19 pandemic [4,5,6,7]. Therefore, we sought to investigate the incidence, mortality (counts and age-standardized rates), and mortality-to-incidence ratio of TBL cancer using the latest IARC estimates for the year 2022 [5]. The main objectives of this study were as follows: (1) to examine the global TBL cancer burden (incidence and mortality) across 21 regions and 185 countries; (2) assess the mortality-to-incidence ratio (MIR) as a proxy for 5-year survival rates across regions and countries; (3) analyze TBL cancer burden in relation to a country’s per capita income and development status, measured by the Human Development Index (HDI); (4) explore male–female disparities in TBL cancer burden across regions and countries; and (5) evaluate the TBL cancer burden with regards to adult tobacco use prevalence and HDI levels.

## 2. Materials and Methods

Data for this study were drawn from GLOBOCAN 2022 estimates of IARC that use data from cancer registries across the world [5]. We utilized estimates pertaining to code C33-34 of the International Classification of Disease (10th version). IARC produced GLOBOCAN estimates by first generating incidence and mortality rates using cancer registry data- population-based, local, and neighboring countries—as per data availability in different countries and by applying short-term prediction models and the mortality-to-incidence ratio [6]. The final estimates of incidence and mortality rates were multiplied with population data from the United Nations Development Program (UNDP). The age-standardized rates were generated by GLOBOCAN using the world standard population proposed by Segi and Doll [6].

Sex differences were highlighted using the age-standardized rate ratio in each region: incidence rate ratio (IRR) and mortality rate ratio (MRR). The IRR and MRR were calculated using the following formula.

$$IRR=\frac{{ASIR}_{males}}{{ASIR}_{females}}$$

$$MRR=\frac{{ASMR}_{males}}{{ASMR}_{females}}$$

The relationship between human development and age-standardized rates of incidence and mortality and MIR was explored using linear regression.

(1) y=α+βx

In the above equation, y is the dependent variable (ASIR, ASMR, MIR), and x is the explanatory variable (alternatively, per capita GDP and HDI 7). The data pertaining to GDP per capita and PPP (constant 2017 international $) were acquired from the World Development Indicators (WDI) database of the World Bank.

We also tested whether tobacco use prevalence is associated with lung cancer burden or not by adding adult tobacco use prevalence as one of the explanatory variables in Equation (1). In the WDI database, tobacco use has been described as the percentage of the population that currently uses any tobacco product (smoked and/or smokeless tobacco) on a daily or non-daily basis. Tobacco products include cigarettes, pipes, cigars, cigarillos, waterpipes (hookah, shisha), bidis, kretek, heated tobacco products, and all forms of smokeless (oral and nasal) tobacco. Tobacco products exclude e-cigarettes (which do not contain tobacco), “e-cigars”, “e-hookahs”, and “e-pipes”.

$$y=\alpha +\beta_{1}x_{1}+\beta_{2}x_{2}$$

In the above equation, $x_{2}$ is the prevalence of adult tobacco use at the country level, which was drawn from the WDI database. The entire data analysis and visualization was performed using Stata 13.0.

## 3. Results

### 3.1. Global and Regional Burden of TBL Cancer

Globally, there were 2.48 million incident cases and 1.82 million deaths due to TBL cancers in 2022. Males accounted for 63.4% of incident cases (1.57 million) and 67.85% of TBL deaths (1.23 million) in 2022 (Table 1). For both sexes combined, the age-standardized incidence rate (ASIR) was 23.1 per 100,000, with ASIR for males (32.1/100,000) almost double that of females (16.2/100,000). The age-standardized mortality rate (ASMR) for both sexes combined at the global level was 16.8/100,000, with ASMR among males (24.8/100,000) nearly double that of females (9.8/100,000). The MIR at the global level stood at 0.71 and was higher among males (0.77) than females.

Eastern Asia shouldered the largest burden of TBL cancers among the 21 UN-defined regions, with around 51% of incident cases (1.24 million) and 46.9% (851,876) of global deaths (851,876) (Table 1). Northern America (incidence: 257,284; deaths: 150,675) and Eastern Europe (incidence: 158,141; deaths: 126,840) were the second and third leading regions for the burden of TBL cancers among both sexes combined in 2022. ASIR was the highest in Eastern Asia (39.4/100,000), followed by Polynesia (37.5/100,000), and was the lowest in African regions (Western Africa: 2.1/100,000; Middle Africa: 2.3/100,000). The ASMR ranged from 2.0/100,000 in Western Africa to 31.7/100,000 in Polynesia. The MIR ranged from 0.54 in Northern America to 0.97 in Micronesia. The MIR ranged between 0.5 and 0.7 in regions of America and Europe, whereas in the low and middle-income regions in Asia, Africa, and America, the MIR was more than 0.7 in most regions (Table 1).

### 3.2. Country-Wise Burden of TBL Cancers

China was the leading country for TBL cancer burden, with 1.06 million new cases and 733,291 deaths in 2022, followed by the United States (new cases: 226,033; deaths: 127,653) (Supplementary Table S2). The top five countries for incident cases accounted for 63.5% of TBL incident cases and 59.0% of TBL deaths in 2022 (i.e., China, the United States, Japan, India, and Russia). The age-standardized rates varied significantly across countries from less than 1/100,000 in Niger (ASIR: 0.95; ASMR: 9.93) to Hungary (ASIR: 47.6; ASMR: 39.8). The country with the highest burden China also had a high ASIR (40.8/100,000) whereas its ASMR was much lower (26.7/100,000). MIR varied from 0.47 in Japan and 0.52 in the United States to 1.0 in several countries.

### 3.3. Gender-Specific Differences in TBL Cancer

**Figure 1:**
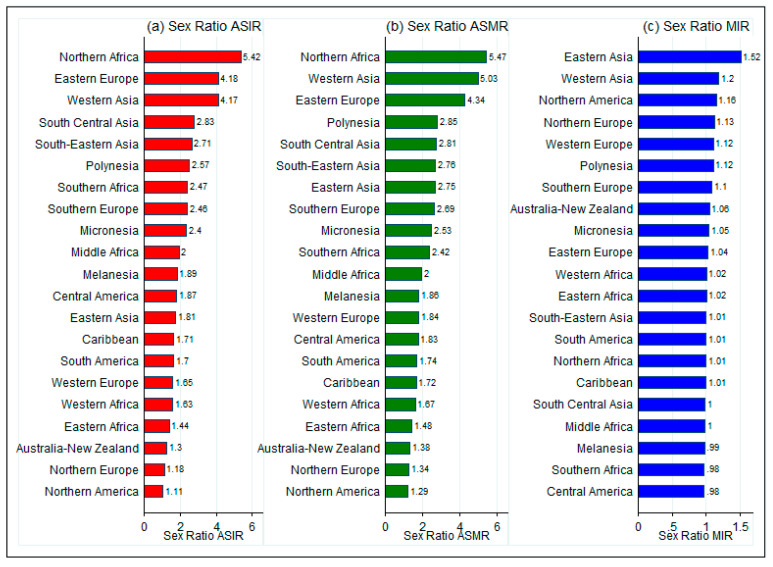
Region-wise sex ratio of TBL cancer burden in 2022. ASIR: Age-standardized Incidence Rate; ASMR: Age-standardized Mortality Rate; MIR: Mortality-to-incidence ratio. MIR was calculated as the ratio of ASMR and ASIR. Source: Authors’ calculations using data from GLOBOCAN 2022. Available online: https://gco.iarc.fr/today/online-analysis-table, accessed on 30 October 2024.

Male–female differences in TBL cancer burden were much starker for Asia and Africa, whereas the male–female disparities were lower in America and Europe (Figure 1). We observed that IRR was as high as 5.42 in Northern Africa, 4.18 in Eastern Europe, and as low as 1.11 in Northern America and 1.3 in Australia/New Zealand. Similar differences in MRR were observed across regions, indicating that male incidence and mortality rates were substantially higher than females in most regions. For MIR, the differences between males and females were not major. Lastly, male–female differences were also visible across countries in terms of age-standardized rates (Supplementary Table S1).

### 3.4. Age-Wise Burden of TBL Cancer

**Figure 2:**
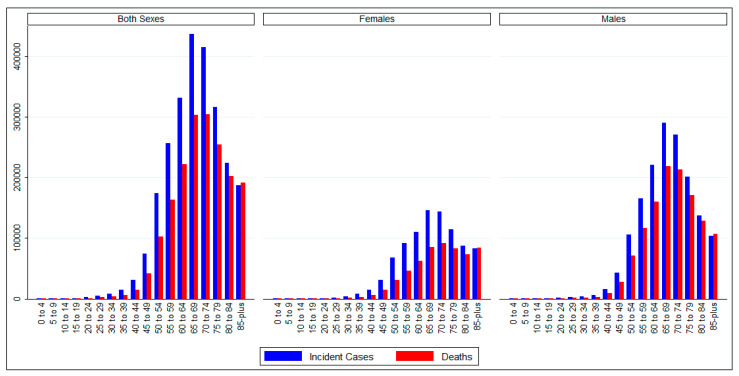
Age-wise counts of Tracheal, Bronchus, and Lung cancer at the global level by sex. Data source: GLOBOCAN 2022 (International Agency for Research on Cancer).

Figure 2 shows the age-wise distribution of TBL cancer incidence and deaths for both sexes combined. In both sexes combined, as well as individual sexes, there were a small number of incident cases and deaths in the below-45 years age groups. Incident cases and deaths peaked in the 65–69 years age group and declined thereafter. Still, a large proportion of cases and deaths occurred in 65-plus years age groups. In most age groups, incident cases and deaths among males were higher than among females.

**Figure 3:**
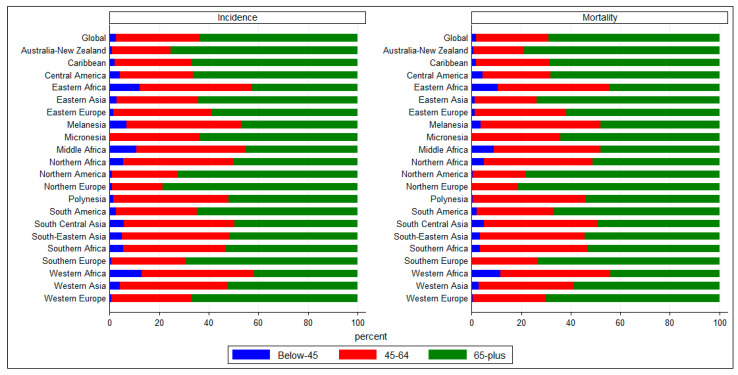
Age-wise composition (%) of TBL cancer burden by regions. Data source: GLOBOCAN 2022 (International Agency for Research on Cancer).

To further explore age-wise distributions in the regions, we grouped age-wise incident cases and death counts into three age groups: below 45 years, 45–65 years, and above 65 years at the regional levels and computed deaths and incident cases occurring in these three broad age-groups (Figure 3). It shows that at the global level, nearly 60% of incident cases and nearly 65% of deaths occurred in those above the age of 65 years. In most regions, a very small percentage of incident cases and deaths occurred in below-45 years age groups. In African regions, a significant percentage of TBL cancers occurred in younger age groups. Cumulatively, below-65 age groups accounted for 55–60% of incident cases and deaths in African regions, whereas in regions of Europe, 65–70% of incident cases and deaths occurred in those above 65 years of age.

### 3.5. Statistical Analysis

We also explored how age-standardized rates across countries are related to country-level per capita income and human development. First, we conducted bivariate association tests between age-standardized rates, MIR, and GDP per capita at the country level (Supplementary Table S2). First, we considered only per capita GDP as the explanatory variable and observed that per capita GDP had a positive association with ASIR and ASMR but a negative association with MIR, implying that age-standardized rates are higher in countries with higher per capita income and survival rates are also higher (i.e., lower MIR) in countries with higher per capita GDP. Per capita GDP could explain 45%, 38%, and 39% of the variation in ASIR, ASMR, and MIR, respectively. Next, we replaced GDP per capita with HDI (often considered a better indicator of development than per capita income). Similar associations with ASIR, ASMR, and MIR were observed, but HDI could explain more variations in ASIR (54%), ASMR (47%), and MIR (48%) than per capita GDP.

Given that smoking is the strongest risk factor for lung cancer, we added adult tobacco use prevalence as an explanatory variable in addition to HDI, and the results are presented in model III (Supplementary Table S2). We observed that adult tobacco use prevalence is statistically significantly associated with ASIR, ASMR, and HDI. Moreover, the explaining power of model III increases with HDI and adult tobacco use prevalence data, explaining a 67% and 64% variation in ASIR and ASMR. However, adult tobacco use prevalence was not statistically significant in case the dependent variable was MIR along with HDI.

**Figure 4:**
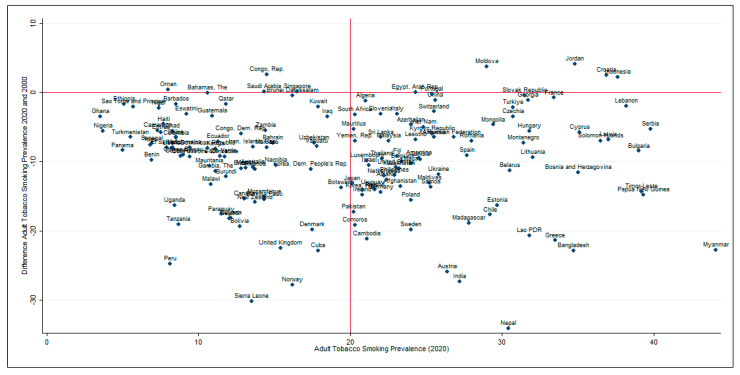
Country-wise adult tobacco smoking prevalence in 2000 and 2020. The vertical axis shows the difference between adult tobacco use prevalence (%) between the years 2020 and 2000. The horizontal axis shows adult tobacco use prevalence in 2020. Data source: World Development Indicators Database of World Bank.

Next, we examined the adult smoking prevalence in 155 countries in 2020 and examined how much the prevalence of adult tobacco use changed between 2000 and 2020 (Figure 4). The horizontal red line describes whether adult tobacco use prevalence has changed between 2000 and 2020; the countries above the horizontal red lines are the countries in which adult tobacco use prevalence has increased between 2000 and 2020. The countries on the right side of the vertical red line are the countries in which adult tobacco use prevalence is more than 20% in 2020. Notably, only a few countries have witnessed an increase in tobacco use prevalence between 2000 and 2020, whereas most countries have witnessed a reduction in the last two decades. Still, there are 81 countries out of 155 where adult tobacco use prevalence was greater than 20% in 2020.

## 4. Discussion

With the help of recently released data by IARC, we investigated the burden of TBL cancers worldwide, focusing on gender and age-wise burden of TBL cancer at global and regional levels. We observed that the TBL cancer burden is highly concentrated in a few countries, with the top five countries accounting for 63.5% of incidence and 59.0% of deaths in 2022. Males have a disproportionately higher TBL cancer burden than females, reflecting the male-to-female difference in tobacco use prevalence across regions. In most regions, older age groups accounted for the highest TBL cancer incidence and deaths, potentially indicating that smoking during adult years may manifest as lung disease after many years.

Eastern Asia leads in the TBL cancer burden, accounting for 51% of global cases and 46.9% of TBL deaths in 2022. China is the leading country in TBL cancer incidence and mortality, with significantly high rates. Previous research has highlighted China’s leading position in the TBL cancer burden [1,8]. While incidence and mortality rates have been falling in other major Western economies (e.g., the United States), they continue to rise in China [9,10]. A recent WHO report on smoking trends indicated that Southeast Asia has the highest percentage of tobacco users (26.5%), followed by the WHO European region (25.3%) [11]. Another recent study highlighted that nearly 300 million Chinese people smoke, with male prevalence around 50% and much higher compared to countries like the United States [12,13]. Several factors contribute to the high smoking prevalence in China, including cultural reasons, low smoking tax, lax warning labels, and weaker state commitment to reducing smoking rates [13]. Besides tobacco smoking, the higher TBL cancer burden in China can also be attributed to worsening air pollution and other environmental carcinogens [14,15].

Apart from China, some countries in the Americas and Europe also are among the highest TBL cancer rates, possibly due to higher smoking prevalence in these specific countries. We also found that the higher the income levels of the country, the greater the lung cancer incidence rates. In the United States, approximately 80% of lung cancer deaths are attributable to cigarette smoking [4]. As smoking rates have peaked, especially among males, lung cancer incidence has been declining since 2006, faster among males (2.5% per annum) than females (1%), possibly due to females starting smoking later and being slower to quit than males [4]. Our analysis also indicated that despite worldwide efforts directed towards tobacco use prevention and control, 81/155 countries had tobacco use prevalence greater than 20% in 2020. These global disparities underscore the urgent need for ongoing efforts to reduce smoking, combat air pollution, and enhance TBL cancer screening.

We also observed stark sex differences in TBL cancer burden, with incidence rate ratios (male:female) as high as 4–5 in some regions. These differences can be partly explained by varying tobacco use prevalence between genders. In developed regions like America and Europe, male and female incidence rates have greater similarity as compared to Asia and Africa, where female tobacco smoking rates are much lower. The elevated sex ratio of age-standardized rates in North Africa warrants further, in-depth exploration. The stark male-to-female disparity in this region can largely be attributed to gender-specific smoking behaviors. Men in North Africa tend to have a much higher smoking prevalence than women, contributing to a disproportionate TBL cancer burden [16]. Cultural norms and socio-economic factors may also play a role, as women often have less exposure to smoking but may still suffer from high rates of lung cancer due to passive smoking or indoor air pollution (particularly from household use of solid fuels for cooking in rural areas) [17]. Moreover, the lack of widespread healthcare access and screening services for women in these regions may result in underreporting of female cases, skewing the sex ratio even further [15,16,17]. Such disparities necessitate targeted public health interventions aimed at reducing smoking rates among men and addressing environmental risk factors among women.

In contrast, sex disparities are not that stark in high-income regions. In high-income countries, smoking rates among females have continued to increase, necessitating efforts to curb smoking rates among females. In the United States, there is evidence of a convergence of lung cancer rates between males and females [18]. It may have happened due to declining smoking rates in males and increasing rates in females; even in a few age groups, smoking rates are becoming similar or higher in females than males [18,19]. The same phenomenon is being observed in European countries where smoking rates and lung cancer burden have been rising in females faster than in males [20]. Research and policy efforts must focus on understanding the rising smoking prevalence among females in some high-income countries. A study in five European countries found that the mean age of smoking initiation among females was 18.2 years, with more than 80% starting before 20 years old [20]. The researchers have identified that 62.3% of ever-smokers reported that they initiated smoking because their friend is a smoker, and more importantly, those who began smoking at younger ages quoted the reason that it looked “cool” [20]. Education significantly predicts smoking habits in the European Union, suggesting that efforts should target discouraging smoking among the less-educated populations along with policy efforts directed towards the reduction of tobacco use [21].

The 5-year survival rates reflected by MIR for TBL cancer are among the lowest for frequently occurring cancers. A cross-country study found the highest 5-year survival rates in Japan (32.9%) and less than 10% in countries like Thailand, Brazil, Bulgaria, and India [22]. In countries such as Japan, the age-standardized incidence and mortality have been higher, but survival rates are notably higher than in other regions and countries. Japan’s superior 5-year survival rate (32.9%) compared to other countries highlights the critical role of early screening programs and access to innovative treatments, such as immunotherapies, which improve patient outcomes. The lower mortality-to-incidence ratio (MIR) in developed nations, such as Japan and the United States, reflects the availability of high-quality healthcare, early diagnosis, and greater access to treatment modalities that are often unavailable in low- and middle-income countries [18,19,20,21,22]. In the U.S., the five-year survival rate for lung cancer has increased significantly, reflecting improvements in screening and treatment modalities, particularly among underserved populations [16,17,18,19]. In contrast, countries with weaker healthcare systems face higher mortality despite similar incidence rates, largely due to late diagnoses and limited access to advanced treatments.

In recent times, immunotherapy-based treatment regimens have shown some promise in boosting the survival rates of patients with NSCLC [23]. Randomized clinical trials found higher survival rates of advanced nonsquamous NSCLC with Nivolumab against Dorcetacel [24] and squamous NSCLC [25]. In an alternate study, however, Nivolumab could not statistically improve progression-free survival among stage IV patients or patients with NSCLC with PD-L1 expression of 5% or more [26]. Low-dose computed tomography (CT) screening reduces lung cancer mortality by 20% [27,28]. Both USPSTF and ACS recommend CT screening for high-risk individuals aged 50 and above with a smoking history of over 20 pack years [29,30]. In China, LDCT screening is recommended for high-risk individuals aged 50–74 years [31]. However, screening uptake rates for lung cancer are lower than for other cancers in the countries with the highest burden of TBL cancers [32].

Beyond tobacco use, ambient air pollution is another major risk factor for lung cancer. As per WHO, fine particles with smaller diameters (<2.5 μm), termed PM2.5, can penetrate deeper into the lungs, causing various pathologies, including lung cancer. Lung cancer rates among non-smokers are higher in polluted areas compared to less polluted areas [33]. In particular, NSCLC with adenocarcinoma histology is characterized by epidermal growth factor receptor (EGFR) mutations, and it generally occurs more in non-smokers, females, and persons belonging to the Asia-Pacific region [34]. A recent study found a significant association between PM2.5 levels and EGFR-driven lung cancer, which is common among non-smokers and light smokers [33]. In 2021, WHO lowered the recommended annual PM2.5 level from 10 µg/m^3^ to 5 µg/m^3^ [35]. All 179 countries for which PM2.5 data were available in 2019 exceeded this recommended level, highlighting the urgent need for countries to reduce PM2.5 levels to lower future TBL cancer burden.

The results of this analysis should be viewed in light of a few potential limitations. First, we utilized secondary data, and our analyses were limited by the variables available in the dataset. Second, given the cross-sectional nature of the study, cause-and-effect relationships cannot be established. Third, the accuracy of GLOBOCAN estimates depends crucially on data availability from cancer registries. Given that estimates for several countries, particularly low and middle countries, were derived from local registries or registries of neighboring countries, the estimates for several low and middle-income countries might be biased. Therefore, policy efforts to establish population-based registries or expand the coverage of existing registries must continue or receive renewed attention. Fourth, some variables need better specification to establish links between exposures and outcomes. For example, tobacco use prevalence in global databases like the ones we used often includes smokeless tobacco use, which may not directly relate to TBL cancers. Despite these limitations, this is the latest and largest estimation of TBL cancers after the COVID-19 pandemic, indicating a need for sustained efforts in reducing one of the deadliest cancers and reasons for premature mortality and greater morbidity.

## 5. Conclusions

In summary, with 1.9 million deaths in 2022, TBL cancer represents a significant global burden. Despite the knowledge of smoking as a risk factor for lung cancer, adult tobacco use prevalence rates remain above 20% in 81 out of 155 countries as of 2019. To reduce smoking prevalence to less than 10% in the future, a renewed global focus and commitment are essential. Specifically, countries bearing the highest global burden must intensify their efforts towards the FTCC and implement new strategies to lower tobacco smoking rates and reduce the TBL cancer burden. Additionally, PM2.5 levels are worsening worldwide, particularly in low- and middle-income countries, potentially reversing the gains made from reduced tobacco smoking. Policymakers at global, regional, and national levels must prioritize further reducing smoking rates and containing rising PM2.5 levels, especially in low- and middle-income countries.

## Figures and Tables

**Table 1:** Burden of Tracheal, Bronchus, and Lung cancer in 2022, by sex.

	Incidence						Deaths						Mortality to Incidence Ratio		
	Count			Age-Standardized Rate			Count			Age-Standardized Rate					
Label	Both	Males	Females	Both	Males	Females	Both	Males	Females	Both	Males	Females	Both	Males	Females
Australia–New Zealand	16,222	9012	7210	24.6	28	21.6	11,313	6406	4907	16.2	19	13.8	0.66	0.68	0.64
Caribbean	11,768	6993	4775	17.9	23.1	13.5	9809	5823	3986	14.8	19.1	11.1	0.83	0.83	0.82
Central America	10,963	6655	4308	5.4	7.3	3.9	10,103	6103	4000	4.9	6.6	3.6	0.91	0.9	0.92
Eastern Africa	7549	4094	3455	3.2	3.9	2.7	6974	3795	3179	3	3.7	2.5	0.94	0.95	0.93
Eastern Asia	1,243,931	783,928	460,003	39.4	51.4	28.4	851,876	596,933	254,943	25.1	37.7	13.7	0.64	0.73	0.48
Eastern Europe	158,147	116,798	41,349	27.6	49.8	11.9	126,840	93,752	33,088	21.6	39.5	9.1	0.78	0.79	0.76
Melanesia	894	569	325	11.6	15.3	8.1	776	494	282	10.2	13.4	7.2	0.88	0.88	0.89
Micronesia	190	127	63	31.6	46.1	19.2	185	125	60	30.5	45.2	17.9	0.97	0.98	0.93
Middle Africa	2055	1311	744	2.3	3.2	1.6	1904	1211	693	2.2	3.6	1.5	0.96	0.97	0.94
Northern Africa	26,280	21,829	4451	11.8	20.6	3.8	23,694	19,734	3960	10.6	18.6	3.4	0.90	0.93	0.89
Northern America	257284	128289	128995	31.9	33.8	30.4	150,675	78,832	71,843	17.2	19.6	15.2	0.54	0.58	0.50
Northern Europe	73,608	37,931	35,677	28	30.6	25.9	52,805	28,107	24,698	18.7	21.7	16.2	0.67	0.71	0.63
Polynesia	287	204	83	37.5	54.7	21.3	244	178	66	31.7	47.6	16.7	0.85	0.87	0.78
South America	82,575	48,338	34,237	13.8	17.9	10.5	70,934	41,559	29,375	11.7	15.3	8.8	0.85	0.85	0.84
South Central Asia	129,889	95,290	34,599	6.6	9.9	3.5	118,183	86,608	31,575	6.1	9.3	3.2	0.92	0.91	0.91
South-Eastern Asia	131,184	91,251	39,933	17.1	26	9.6	116,366	80,989	35,377	15.2	23.2	8.4	0.89	0.89	0.88
Southern Africa	9696	6201	3495	16.8	25.7	10.4	8904	5662	3242	15.7	23.7	9.8	0.93	0.92	0.94
Southern Europe	106,610	73,348	33,262	27.7	40.8	16.6	86,190	60,598	25,592	21.0	31.8	11.8	0.76	0.78	0.71
Western Africa	4251	2420	1831	2.1	2.6	1.6	3988	2291	1697	2.0	2.5	1.5	0.95	0.96	0.94
Western Asia	61,351	48,464	12,887	23.3	38.8	9.3	55,972	45,492	10,480	21.5	37.2	7.4	0.92	0.96	0.8
Western Europe	145,941	88,993	56,948	31.2	39.6	24	109,734	68,549	41,185	22.1	29.3	15.9	0.71	0.74	0.66
Global	2,480,675	1,572,045	908630	23.6	32.1	16.2	1,817,469	1,233,241	584,228	16.8	24.8	9.8	0.71	0.77	0.69

Data source: GLOBOCAN 2022.

## Data Availability

All the data are available online: https://gco.iarc.fr/today/online-analysis-table, accessed on 30 October 2024.

## Supplementary Materials

The following supporting information can be downloaded at: https://www.mdpi.com/article/10.3390/epidemiologia5040053/s1; Table S1: Country-wise Burden of Tracheal, Bronchus and Lung Cancer in 2022; Table S2: Association between TBL Cancer Burden, HDI and Adult Tobacco Prevalence.
