# Supplementary material for: Global, Regional, and National Burden of Tracheal, Bronchus, and Lung Cancer in 2022: Evidence from the GLOBOCAN Study
Source: Epidemiologia (Basel). 2024 Dec 17;5(4):785–95. doi: 10.3390/epidemiologia5040053 (PMC11675692; doi:10.3390/epidemiologia5040053)
Supplement: Supplementary file 1 [file epidemiologia-05-00053-s001.zip › epidemiologia-3220687-supplementary.pdf]

Supplementary Table S1. Country-wise Burden of Tracheal, Bronchus and Lung Cancer in 2022

	Incidence						Mortality						Mortality-to-incidence ratio		
	Count			Age-standardized Rate			Count			Age-standardized Rate					
**Country**	**Both**	**M**	**F**	**Both**	**M**	**F**	**Both**	**M**	**F**	**Both**	**M**	**F**	**Both**	**M**	**F**
Afghanistan	1393	1019	374	7.2	11	3.6	1318	977	341	6.8	10.3	3.4	0.94	0.94	0.94
Albania	1438	1134	304	25.3	41.5	10.3	1264	997	267	24.2	39.9	9.6	0.96	0.96	0.93
Algeria	5040	4311	729	11.3	19.5	3.2	4599	3943	656	10.4	17.9	2.9	0.92	0.92	0.91
Angola	338	223	115	2.4	3.3	1.6	309	200	109	2.2	3	1.5	0.92	0.91	0.94
Argentina	13016	8587	4429	19.9	30	12.1	10673	7032	3641	16.1	24.3	9.8	0.81	0.81	0.81
Armenia	1428	1145	283	29	57.4	9.1	1187	952	235	23.7	47.2	7.5	0.82	0.82	0.82
Australia	13426	7353	6073	24.1	27.1	21.5	9191	5271	3920	15.6	18.5	13	0.65	0.68	0.6
Austria	5410	3004	2406	27.2	31.2	23.9	4259	2506	1753	19.5	24.3	15.4	0.72	0.78	0.64
Azerbaijan	2350	2004	346	19.4	37.5	5.2	1954	1665	289	16.2	31.4	4.3	0.84	0.84	0.83
Bahamas	45	30	15	8.8	13.1	5.3	36	25	11	7	10.9	3.8	0.8	0.83	0.72
Bahrain	89	64	25	10.1	15.1	5.3	77	56	21	8.6	12.9	4.7	0.85	0.85	0.89
Bangladesh	13000	10033	2967	8.8	13.5	4	12063	9313	2750	8.2	12.5	3.7	0.93	0.93	0.93
Barbados	57	33	24	9.4	13.2	6	46	26	20	7.5	10.1	5.1	0.8	0.77	0.85
Belarus	4599	4006	593	26	56.5	5.5	3824	3363	461	21.1	47.1	3.9	0.81	0.83	0.71
Belgium	8636	5469	3167	32.7	42.3	24.4	6102	3888	2214	21.4	28.3	15.6	0.65	0.67	0.64
Belize	28	21	7	8.8	13.6	4	26	19	7	8.1	12.2	4	0.92	0.9	1

	Incidence						Mortality						Mortality-to-incidence ratio		
	Count			Age-standardized Rate			Count			Age-standardized Rate					
**Country**	**Both**	**M**	**F**	**Both**	**M**	**F**	**Both**	**M**	**F**	**Both**	**M**	**F**	**Both**	**M**	**F**
Benin	104	60	44	1.5	2.1	1.1	94	55	39	1.4	2	1	0.93	0.95	0.91
Bhutan	51	26	25	7	6.5	7.6	51	26	25	7	6.5	7.6	1	1	1
Bolivia	1163	769	394	8.5	11.6	6	1001	663	338	7.3	9.9	5.1	0.86	0.85	0.85
Bosnia Herzegovina	2436	1862	574	36.1	59.5	15.7	2002	1528	474	28.2	46.9	12.3	0.78	0.79	0.78
Botswana	63	46	17	3.6	6.5	1.7	59	42	17	3.4	6	1.7	0.94	0.92	1
Brazil	44213	24804	19409	14.6	18.3	11.7	38292	21515	16777	12.5	15.7	9.9	0.86	0.86	0.85
Brunei Darussalam	108	57	51	24	25.6	22.6	91	51	40	21.1	23.8	18.4	0.88	0.93	0.81
Bulgaria	3966	2916	1050	27.1	43.8	13.3	3435	2538	897	22.9	37.2	11.1	0.85	0.85	0.83
Burkina Faso	351	99	252	3.8	3	4.5	340	97	243	3.7	3	4.4	0.97	1	0.98
Burundi	182	97	85	3.4	4.2	2.8	167	90	77	3.2	4	2.6	0.94	0.95	0.93
Cambodia	2161	1374	787	15.7	23.9	9.9	1913	1219	694	14	21.4	8.8	0.89	0.9	0.89
Cameroon	393	259	134	3.1	4.4	1.9	366	241	125	2.9	4.2	1.9	0.94	0.95	1
Canada	31157	15900	15257	32.4	34.9	30.4	22970	11959	11011	21.5	24.4	19.1	0.66	0.7	0.63
Cape Verde	9	3	6	2.2	1.8	2.5	7	3	4	1.5	2	1.4	0.68	1.11	0.56
Central African Republic	46	28	18	2	2.7	1.5	44	26	18	2	2.6	1.5	1	0.96	1
Chad	159	134	25	2	3.5	0.64	153	129	24	1.9	3.4	0.62	0.95	0.97	0.97
Chile	4391	2619	1772	12.7	17	9.2	3944	2348	1596	11.3	15.1	8.2	0.89	0.89	0.89

	Incidence						Mortality						Mortality-to-incidence ratio		
	Count			Age-standardized Rate			Count			Age-standardized Rate					
**Country**	**Both**	**M**	**F**	**Both**	**M**	**F**	**Both**	**M**	**F**	**Both**	**M**	**F**	**Both**	**M**	**F**
China	1060584	658722	401862	40.8	52	30.3	733291	515909	217382	26.7	39.5	14.7	0.65	0.76	0.49
Colombia	7196	4178	3018	10.1	13.1	7.6	6124	3532	2592	8.5	11	6.5	0.84	0.84	0.86
Comoros	9	6	3	1.8	2.6	1.1	9	6	3	1.8	2.6	1.1	1	1	1
Congo, Democratic People Republic of	998	594	404	2.2	2.8	1.6	920	549	371	2	2.6	1.5	0.91	0.93	0.94
Congo, Republic of	40	27	13	1.4	2.1	0.8	36	24	12	1.3	1.9	0.75	0.93	0.9	0.94
Costa Rica	527	360	167	6.8	9.7	4.1	400	253	147	4.9	6.6	3.4	0.72	0.68	0.83
Côte d'Ivoire	338	243	95	2.5	3.5	1.4	293	214	79	2.2	3.2	1.2	0.88	0.91	0.86
Croatia	3649	2492	1157	37.1	56.4	21.6	2951	2063	888	29.6	46.3	16.3	0.8	0.82	0.75
Cuba	7339	4426	2913	29.8	38.3	22.4	6066	3659	2407	24.3	31.4	18.1	0.82	0.82	0.81
Cyprus	625	456	169	27	41.4	14.6	532	394	138	22.4	35.5	10.8	0.83	0.86	0.74
Czechia	6192	3709	2483	23.7	31.3	17.6	5006	3120	1886	19.8	27.3	13.5	0.84	0.87	0.77
Denmark	4973	2408	2565	34.2	34.3	34.4	3738	1896	1842	24.5	25.7	23.6	0.72	0.75	0.69
Djibouti	26	16	10	3.1	3.6	2.5	22	14	8	2.6	3.3	1.9	0.84	0.92	0.76
Dominican Republic	1620	896	724	12.8	15.1	10.8	1379	764	615	10.9	12.8	9.1	0.85	0.85	0.84
Ecuador	1348	664	684	6.3	6.6	6	1145	602	543	5.3	6	4.8	0.84	0.91	0.8
Egypt	7643	5599	2044	8.8	13.9	4.4	6805	5000	1805	7.9	12.6	3.9	0.9	0.91	0.89
El Salvador	386	182	204	4.7	5.2	4.3	348	169	179	4.2	4.9	3.7	0.89	0.94	0.86

	Incidence						Mortality						Mortality-to-incidence ratio		
	Count			Age-standardized Rate			Count			Age-standardized Rate					
**Country**	**Both**	**M**	**F**	**Both**	**M**	**F**	**Both**	**M**	**F**	**Both**	**M**	**F**	**Both**	**M**	**F**
Equatorial Guinea	20	11	9	2.7	2.9	2.5	19	10	9	2.7	2.8	2.5	1	0.97	1
Eritrea	72	38	34	3.2	3.7	2.8	63	31	32	2.8	3.1	2.6	0.88	0.84	0.93
Estonia	810	563	247	25.8	45	12.6	653	477	176	20.1	37.7	7.7	0.78	0.84	0.61
Eswatini	29	19	10	4.1	7.6	2.4	29	19	10	4.1	7.6	2.4	1	1	1
Ethiopia	2486	1226	1260	3.7	3.9	3.5	2421	1202	1219	3.6	3.8	3.4	0.97	0.97	0.97
Fiji	60	37	23	6.8	9.1	4.9	58	36	22	6.6	8.9	4.6	0.97	0.98	0.94
Finland	3068	1828	1240	19.2	25.1	14.2	2289	1431	858	14.1	19.5	9.5	0.73	0.78	0.67
France (metropolitan)	49613	32812	16801	35.9	49	24.7	36876	24829	12047	24.2	35.1	14.8	0.67	0.72	0.6
France, Guadeloupe	106	71	35	12.3	18.7	7.2	92	65	27	10.5	16.5	5.7	0.85	0.88	0.79
France, La Réunion	347	267	80	21.8	36.3	9.2	299	234	65	18.5	31.1	7.5	0.85	0.86	0.82
France, Martinique	90	55	35	9	12.7	6.1	79	51	28	8	11.8	4.9	0.89	0.93	0.8
French Guyana	43	32	11	15	23.8	7.5	35	26	9	12.3	19.6	6.1	0.82	0.82	0.81
French Polynesia	154	111	43	40	57.4	22.6	136	101	35	35	51.9	18.1	0.88	0.9	0.8
Gabon	45	25	20	3.1	3.5	2.7	41	22	19	2.9	3.1	2.6	0.94	0.89	0.96
Gaza Strip and West Bank	586	485	101	20.4	35.8	6.6	543	453	90	18.9	33.4	6	0.93	0.93	0.91
Georgia	1572	1363	209	22	45.9	4.4	1317	1122	195	18.1	37.5	4	0.82	0.82	0.91
Germany	62025	36871	25154	28.1	35.2	22	47731	29018	18713	21.4	27.4	16.1	0.76	0.78	0.73

	Incidence						Mortality						Mortality-to-incidence ratio		
	Count			Age-standardized Rate			Count			Age-standardized Rate					
**Country**	**Both**	**M**	**F**	**Both**	**M**	**F**	**Both**	**M**	**F**	**Both**	**M**	**F**	**Both**	**M**	**F**
Ghana	519	299	220	2.5	3.1	2	492	290	202	2.4	3.1	1.9	0.96	1	0.95
Greece	8583	6357	2226	31.3	49.6	15.7	7156	5417	1739	24.6	40.1	11.5	0.79	0.81	0.73
Guam	84	55	29	31.5	45.5	19.1	82	54	28	30.3	44.3	17.9	0.96	0.97	0.94
Guatemala	545	293	252	3.8	4.5	3.2	512	275	237	3.5	4.2	3	0.92	0.93	0.94
Guinea	241	160	81	3.2	5.2	1.9	241	159	82	3.2	5.1	1.9	1	0.98	1
Guinea-Bissau	23	15	8	2.1	3.1	1.4	22	15	7	2	3.1	1.2	0.95	1	0.86
Guyana	37	26	11	4.3	6.4	2.4	34	23	11	3.8	5.5	2.4	0.88	0.86	1
Haiti	561	232	329	6.1	5.5	6.6	509	222	287	5.5	5.3	5.7	0.9	0.96	0.86
Honduras	518	312	206	6.7	8.8	4.9	392	197	195	4.9	5.4	4.5	0.73	0.61	0.92
Hungary	9911	5706	4205	47.6	64.4	35	8462	4890	3572	39.8	54.5	28.9	0.84	0.85	0.83
Iceland	196	92	104	26.9	26.1	27.8	121	57	64	15.4	15.8	15	0.57	0.61	0.54
India	81748	58970	22778	5.8	8.5	3.2	75031	54220	20811	5.3	7.8	2.9	0.91	0.92	0.91
Indonesia	38904	29107	9797	13.4	21.3	6.4	34339	25589	8750	11.9	19	5.7	0.89	0.89	0.89
Iran, Islamic Republic of	10934	7576	3358	12.1	16.7	7.4	9895	6781	3114	11	14.9	7	0.91	0.89	0.95
Iraq	2814	2364	450	12.5	23.5	3.6	2613	2201	412	11.6	21.8	3.3	0.93	0.93	0.92
Ireland	3086	1557	1529	31.4	32.6	30.4	2020	1051	969	19.4	21.5	17.5	0.62	0.66	0.58
Israel	3006	1818	1188	22.6	29.9	16.1	1958	1218	740	13.6	18.9	8.9	0.60	0.63	0.55

	Incidence						Mortality						Mortality-to-incidence ratio		
	Count			Age-standardized Rate			Count			Age-standardized Rate					
**Country**	**Both**	**M**	**F**	**Both**	**M**	**F**	**Both**	**M**	**F**	**Both**	**M**	**F**	**Both**	**M**	**F**
Italy	43808	28450	15358	24.6	34.5	16.2	35668	23608	12060	18	25.9	11.6	0.73	0.75	0.72
Jamaica	545	350	195	14	19	9.4	461	297	164	11.8	16.1	7.9	0.84	0.85	0.84
Japan	136723	95740	40983	30.5	47.2	15.8	83243	56905	26338	14.2	22.7	7.1	0.47	0.48	0.45
Jordan	1048	853	195	14.3	24.1	5.1	967	791	176	13.3	22.5	4.7	0.93	0.93	0.92
Kazakhstan	4429	3822	607	19.7	41.4	4.5	3688	3142	546	16.4	34.1	4.1	0.83	0.82	0.91
Kenya	903	421	482	3.4	3.6	3.2	822	382	440	3.1	3.3	3	0.91	0.92	0.94
Korea, Democratic People Republic of	14773	7865	6908	38.4	50.1	29.8	12432	6506	5926	32.1	42.1	25	0.84	0.84	0.84
Korea, Republic of	31337	21183	10154	26.2	38.6	16.4	22474	17258	5216	16.7	30	6.5	0.64	0.78	0.4
Kuwait	207	155	52	6.4	7.7	4.3	178	136	42	5.8	7	3.8	0.91	0.91	0.88
Kyrgyzstan	782	597	185	14.1	26.3	5.7	636	466	170	11.4	20.2	5.3	0.81	0.77	0.93
Lao People's Democratic Republic	961	695	266	17.6	27.1	9.1	853	626	227	15.6	24.3	7.9	0.89	0.9	0.87
Latvia	1018	776	242	23.5	45.7	9.2	878	662	216	19.7	39	6.9	0.84	0.85	0.75
Lebanon	1566	1004	562	19.9	25.7	14.3	1408	905	503	17.8	23.1	12.7	0.89	0.9	0.89
Lesotho	68	31	37	4.2	5	3.7	61	28	33	3.8	4.6	3.3	0.90	0.92	0.89
Liberia	88	63	25	2.7	4	1.5	83	58	25	2.6	3.8	1.5	0.96	0.95	1
Libya	934	836	98	16.4	32.2	3.2	857	766	91	15.2	29.9	3	0.93	0.93	0.94

	Incidence						Mortality						Mortality-to-incidence ratio		
	Count			Age-standardized Rate			Count			Age-standardized Rate					
**Country**	**Both**	**M**	**F**	**Both**	**M**	**F**	**Both**	**M**	**F**	**Both**	**M**	**F**	**Both**	**M**	**F**
Lithuania	1494	1104	390	24.2	44.9	10.4	1241	934	307	19.8	38	7.3	0.82	0.85	0.7
Luxembourg	360	210	150	25.7	31.6	21	268	158	110	20.7	25.3	16.6	0.81	0.8	0.79
Madagascar	335	200	135	2.1	2.8	1.5	305	186	119	1.9	2.6	1.4	0.90	0.93	0.93
Malawi	138	86	52	1.6	2.2	1	129	79	50	1.5	2.1	0.97	0.94	0.95	0.97
Malaysia	5503	4196	1307	15.1	23.2	7.1	4783	3691	1092	13.1	20.4	5.9	0.87	0.88	0.83
Maldives	47	37	10	12.4	18.1	5.9	42	32	10	11.7	17	5.9	0.94	0.94	1
Mali	295	193	102	3.2	4.5	2	289	189	100	3.1	4.5	2	0.97	1	1
Malta	227	164	63	19.2	27.7	11.9	174	131	43	14.9	22.3	8.6	0.78	0.81	0.72
Mauritania	55	37	18	1.9	2.8	1.2	51	34	17	1.8	2.7	1.1	0.95	0.96	0.92
Mauritius	187	124	63	8.4	12.2	5.3	176	119	57	7.8	11.6	4.5	0.93	0.95	0.85
Mexico	8257	5062	3195	5.4	7.3	3.8	7808	4820	2988	5.1	6.9	3.6	0.94	0.95	0.95
Moldova	1464	1178	286	22.3	42.8	7.1	1263	1022	241	19	37	5.9	0.85	0.86	0.83
Mongolia	514	418	96	19	35.9	6.4	436	355	81	16.4	31.6	5.4	0.86	0.88	0.84
Montenegro	419	310	109	36.7	58.8	17.2	356	257	99	30.2	47.5	15.4	0.82	0.81	0.90
Morocco	8825	7884	941	20.4	38.4	4.2	7970	7136	834	18.5	34.8	3.7	0.91	0.91	0.88
Mozambique	210	142	68	1.3	2.4	0.68	205	139	66	1.3	2.3	0.67	1.00	0.96	0.99
Myanmar	8868	4983	3885	16.1	21.6	12.2	8145	4580	3565	14.8	19.9	11.2	0.92	0.92	0.92
Namibia	90	52	38	5.8	8.3	4.1	83	47	36	5.5	7.8	4.00	0.95	0.94	0.98

	Incidence						Mortality						Mortality-to-incidence ratio		
	Count			Age-standardized Rate			Count			Age-standardized Rate					
**Country**	**Both**	**M**	**F**	**Both**	**M**	**F**	**Both**	**M**	**F**	**Both**	**M**	**F**	**Both**	**M**	**F**
Nepal	2431	1528	903	9.3	12.9	6.3	2207	1389	818	8.4	11.8	5.70	0.90	0.91	0.90
New Caledonia	170	115	55	41.8	59.3	25.8	127	87	40	31	44.7	18.3	0.74	0.75	0.71
New Zealand	2796	1659	1137	27.3	33.1	22.2	2122	1135	987	19.4	21.6	17.7	0.71	0.65	0.80
Nicaragua	332	188	144	5.4	7.1	4.2	311	174	137	5.1	6.6	3.9	0.94	0.93	0.93
Niger	102	97	5	0.95	1.8	0.09	101	96	5	0.93	1.8	0.09	0.98	1.00	1.00
Nigeria	1675	812	863	1.7	1.9	1.6	1561	768	793	1.6	1.8	1.5	0.94	0.95	0.94
North Macedonia	1150	873	277	31.1	49.4	14.3	1002	757	245	26.8	42.6	12.5	0.86	0.86	0.87
Norway	3604	1799	1805	28	28.7	27.6	2497	1257	1240	17.6	18.9	16.6	0.63	0.66	0.60
Oman	157	124	33	5.2	7	2.8	146	116	30	4.9	6.5	2.7	0.94	0.93	0.96
Pakistan	9464	7571	1893	6	9.5	2.3	8307	6635	1672	5.3	8.3	2.1	0.88	0.87	0.91
Panama	370	237	133	6.4	8.7	4.3	306	196	110	5.2	7.1	3.5	0.81	0.82	0.81
Papua New Guinea	621	384	237	10.7	13.6	7.9	553	343	210	9.6	12.4	7.1	0.9	0.91	0.90
Paraguay	910	740	170	12.7	21.4	4.5	772	628	144	10.7	18	3.8	0.84	0.84	0.84
Peru	2919	1443	1476	6.8	6.9	6.7	2630	1316	1314	6	6.2	5.9	0.88	0.9	0.88
Philippines	23728	16588	7140	24	37.7	13	20953	14654	6299	21.3	33.6	11.5	0.89	0.89	0.88
Poland	30379	19206	11173	36.5	52.5	24.1	26035	16596	9439	30.5	44.7	19.7	0.84	0.85	0.82
Portugal	6155	4253	1902	23.9	36.9	13.3	5077	3686	1391	18.5	30.4	8.9	0.77	0.82	0.67
Puerto Rico	761	471	290	10.2	14.9	6.8	608	362	246	7.5	10.8	5.1	0.74	0.72	0.75

	Incidence						Mortality						Mortality-to-incidence ratio		
	Count			Age-standardized Rate			Count			Age-standardized Rate					
**Country**	**Both**	**M**	**F**	**Both**	**M**	**F**	**Both**	**M**	**F**	**Both**	**M**	**F**	**Both**	**M**	**F**
Qatar	92	77	15	5.8	6.1	4.6	81	66	15	5.3	5.5	4.6	0.91	0.9	1.00
Romania	11716	8499	3217	30.3	49.3	14.6	10530	7743	2787	26.6	44.1	12.2	0.88	0.89	0.84
Russian Federation	70362	56078	14284	26	52.4	8.6	51887	41249	10638	18.6	38.1	5.9	0.72	0.73	0.69
Rwanda	224	161	63	2.8	4.4	1.5	204	146	58	2.6	4.1	1.4	0.93	0.93	0.93
Saint Lucia	26	21	5	9.7	16.5	3.4	21	17	4	7.9	13.2	3.0	0.81	0.80	0.88
Samoa	54	38	16	33.3	49.6	18.9	40	28	12	25	37.4	14.1	0.75	0.75	0.75
Sao Tome and Principe	16	10	6	12.7	16	9.5	16	10	6	12.7	16	9.5	1.0	1	1
Saudi Arabia	1217	939	278	4.3	5.6	2.5	1048	815	233	3.9	5	2.2	0.91	0.89	0.88
Senegal	259	175	84	2.7	4.2	1.5	240	163	77	2.5	3.9	1.4	0.93	0.93	0.93
Serbia	6879	4669	2210	40.4	59.6	23.8	5818	3939	1879	33.4	49.4	19.7	0.83	0.83	0.83
Sierra Leone	53	52	1	1.3	2.7	0.02	48	47	1	1.2	2.6	0.02	0.92	0.96	1
Singapore	2940	1981	959	24.3	33.2	15.9	2747	1899	848	22.2	31.9	13.2	0.91	0.96	0.83
Slovakia	2641	1840	801	22.6	36.5	12.3	2283	1597	686	19.3	31.5	10.3	0.85	0.86	0.84
Slovenia	1729	1042	687	34.2	43.8	25.7	1312	799	513	25.4	33.2	18.5	0.74	0.76	0.72
Solomon Islands	28	21	7	6.2	9.4	3.2	23	16	7	5.3	7.6	3.2	0.85	0.81	1
Somalia	271	145	126	3.5	3.9	3.1	252	135	117	3.3	3.7	3	0.94	0.95	0.97
South Africa	9446	6053	3393	18.1	27.6	11.2	8672	5526	3146	16.9	25.5	10.5	0.93	0.92	0.94

	Incidence						Mortality						Mortality-to-incidence ratio		
	Count			Age-standardized Rate			Count			Age-standardized Rate					
**Country**	**Both**	**M**	**F**	**Both**	**M**	**F**	**Both**	**M**	**F**	**Both**	**M**	**F**	**Both**	**M**	**F**
South Sudan	131	72	59	2.1	2.4	1.8	120	65	55	2	2.2	1.7	0.95	0.92	0.94
Spain	30041	21654	8387	27.8	41.4	16	23297	17318	5979	20.3	31.6	10.6	0.73	0.76	0.66
Sri Lanka	2229	1666	563	6.7	11.2	3.1	2035	1520	515	6.1	10.3	2.9	0.91	0.92	0.94
Sudan	673	407	266	2.5	3.1	1.9	623	376	247	2.4	2.9	1.8	0.96	0.94	0.95
Suriname	113	73	40	16.6	24.1	10.4	93	61	32	13.7	20.2	8.3	0.83	0.84	0.8
Sweden	4441	1978	2463	17.4	15.8	19.1	3816	1718	2098	13.3	12.9	13.7	0.76	0.82	0.72
Switzerland	5028	2795	2233	24.1	27.9	20.6	3556	2029	1527	15.9	19.1	13	0.66	0.68	0.63
Syrian Arab Republic	2638	1987	651	17.8	28.7	8.2	2517	1866	651	17.2	27.4	8.1	0.97	0.95	0.99
Tajikistan	346	219	127	5.4	7.1	3.8	309	194	115	4.9	6.4	3.5	0.91	0.9	0.92
Tanzania, United Republic of	795	408	387	2.5	2.8	2.3	693	359	334	2.2	2.5	2	0.88	0.89	0.87
Thailand	23494	15200	8294	18.1	25.9	11.7	19864	12853	7011	15	21.5	9.6	0.83	0.83	0.82
The Netherlands	14806	7793	7013	34.9	36.5	33.9	10896	6091	4805	23.6	26.5	21.3	0.68	0.73	0.63
The Republic of the Gambia	55	50	5	5	9.5	0.87	55	50	5	5	9.5	0.87	1	1	1
Timor-Leste	91	63	28	10.2	14.7	6	81	55	26	9.1	13	5.5	0.89	0.88	0.92
Togo	84	62	22	1.9	3.1	0.88	71	53	18	1.7	2.8	0.76	0.89	0.9	0.86
Trinidad and Tobago	264	199	65	11.7	19.2	5.1	216	161	55	9.5	15.5	4.3	0.81	0.81	0.84

	Incidence						Mortality						Mortality-to-incidence ratio		
	Count			Age-standardized Rate			Count			Age-standardized Rate					
**Country**	**Both**	**M**	**F**	**Both**	**M**	**F**	**Both**	**M**	**F**	**Both**	**M**	**F**	**Both**	**M**	**F**
Tunisia	3108	2742	366	20.1	37.8	4.5	2787	2466	321	18.1	34	3.9	0.9	0.9	0.87
Türkiye	41032	33039	7993	37.9	68	13.5	38505	32119	6386	35.1	66.3	10.1	0.93	0.98	0.75
Turkmenistan	504	372	132	9.5	16.1	4.5	464	352	112	8.7	15.2	3.8	0.92	0.94	0.84
Uganda	562	293	269	3	3.5	2.6	480	253	227	2.6	3.1	2.3	0.87	0.89	0.88
Ukraine	16917	13660	3257	20.6	41.7	6.3	14115	11634	2481	17	35.4	4.6	0.83	0.85	0.73
United Arab Emirates	249	184	65	6.2	6.8	5.1	221	170	51	5.4	5.9	4.4	0.87	0.87	0.86
United Kingdom	50700	25711	24989	30.1	31.9	28.5	35394	18539	16855	19.4	21.8	17.4	0.64	0.68	0.61
United States of America	226033	112343	113690	31.9	33.7	30.4	127653	66846	60807	16.6	19	14.7	0.52	0.56	0.48
Uruguay	1752	1220	532	29.7	46.6	16.4	1574	1140	434	25.4	41.8	12.7	0.86	0.9	0.77
Uzbekistan	2531	1854	677	8.4	13.6	4.1	2137	1561	576	7.1	11.5	3.5	0.85	0.85	0.85
Vanuatu	15	12	3	7.7	11.8	3.4	15	12	3	7.7	11.8	3.4	1	1	1
Venezuela	5474	3183	2291	15.9	20.4	12.2	4617	2673	1944	13.3	17.1	10.3	0.84	0.84	0.84
Viet Nam	24426	17007	7419	20.3	31.5	11	22597	15772	6825	18.7	29.3	10	0.92	0.93	0.91
Yemen	774	443	331	4.1	5	3.2	749	434	315	3.9	4.9	3.1	0.95	0.98	0.97
Zambia	231	131	100	3	4.6	2.2	204	116	88	2.8	4.3	2	0.93	0.93	0.91
Zimbabwe	431	255	176	5.9	9.2	3.9	394	233	161	5.5	8.6	3.6	0.93	0.93	0.92
**Total**	**2479698**	**1571405**	**908293**	**23.6**	**32.1**	**16.2**	**1816600**	**1232641**	**583959**	**16.8**	**24.8**	**9.8**	**0.71**	**0.77**	**0.6**

Supplementary Table S2. Association between TBL Cancer Burden, HDI and Adult Tobacco Prevalence

	Model I			Model II			Model III		
**Dependent Variable→ Independent Variables ↓**	**ASIR**	**ASMR**	**MIR**	**ASIR**	**ASMR**	**MIR**	**ASIR**	**ASMR**	**MIR**
Ln(Per Capita GDP)	6.35*** (0.54)	4.47*** (0.43))	-0.05*** (0.005)						
HDI				52.50*** (3.68)	37.12*** (3.00)	-0.44*** (0.035)	45.82*** (3.68)	30.26*** (2.87)	-0.47*** (0.04)
Adult Tobacco Prevalence							0.43*** (0.06)	0.39*** (0.048)	0.0004 (0.0007)
Intercept	-44.94*** (5.16)	-30.31*** (4.11)	1.37*** (0.05)	-23.06*** (2.71)	-23.06*** (2.71)	1.18*** (0.3)	-26.47*** (2.59)	-17.41*** (2.02)	1.19*** (0.03)
No. of Observations	171	175	154	171	175	154	171	175	154
Adjusted-R-squared	0.45	0.38	0.39	0.54	0.47	0.48	0.67	0.64	0.49

ASIR: Age-standardized Incidence Rate; ASMR: Age-standardized Mortality Rate; MIR: Mortality-to-incidence ratio. MIR was calculated as the ratio of ASMR and ASIR. Source: Authors' calculations using data from GLOBOCAN 2022. */**/*** indicates statistical significance at 10/5/1% levels respectively. The data pertaining to HDI was procured from United Nations Development Program and data pertaining to per capita GDP and adult tobacco prevalence was procured from World Development Indicators database of World Bank.
